# Correction: Exploring midwives’ understanding of respectful maternal care in Kumasi, Ghana: Qualitative inquiry

**DOI:** 10.1371/journal.pone.0238278

**Published:** 2020-08-21

**Authors:** Veronica Millicent Dzomeku, Bemah Adwoa Boamah Mensah, Emmanuel Kweku Nakua, Pascal Agbadi, Jody R. Lori, Peter Donkor

The first author’s name is spelled incorrectly. The correct name is: Veronica Millicent Dzomeku. The correct citation is: Dzomeku VM, Mensah BAB, Nakua EK, Agbadi P, Lori JR, Donkor P (2020) Exploring midwives’ understanding of respectful maternal care in Kumasi, Ghana: Qualitative inquiry. PLoS ONE 15(7): e0220538. https://doi.org/10.1371/journal.pone.0220538.
